# The Impact of Positive Mood and Future Outlook on English as a Foreign Language Students’ Academic Self-Concept

**DOI:** 10.3389/fpsyg.2022.846422

**Published:** 2022-02-11

**Authors:** Jingsheng Zhang

**Affiliations:** School of Foreign Languages, Xinyang College, Xinyang, China

**Keywords:** positive mood, future outlook, academic self-concept, EFL students, positive psychology

## Abstract

Due to the fact that English as a foreign language (EFL) students’ academic self-concept is of high importance for their academic motivation, academic achievement, and L2 success, many investigations have been done to uncover the personal and interpersonal factors that may contribute to students’ academic self-concept. Yet, the emotional and psychological factors have rarely been studied. In addition, no empirical and review study has been carried out to probe into the impact of positive mood and future outlook on EFL students’ academic self-concept. Accordingly, the current review study seeks to illustrate the effects of positive mood and future outlook on EFL students’ academic self-concept. Building upon the principles of positive psychology (PP), the favorable effects of positive mood and future outlook on EFL students’ academic self-concept were thoroughly explained. The educational implications and suggestions for future research are also highlighted.

## Introduction

Students’ thoughts and perceptions of themselves and their academic capabilities have gained momentum in the educational domain due to the undeniable function they serve in understanding what drives them to act or abstain from acting in classroom contexts (Erten and Burden, 2014). The personal thoughts and perceptions of students about themselves and their academic abilities are called “*student academic self-concept*” (De Fraine et al., 2007). According to Marsh and Martin (2011, p. 60), student academic self-concept refers to “students’ personal beliefs of their academic abilities and skills that are developed through experience with and interpreting the learning environment.” It is suggested that how individual students conceive themselves and their abilities can largely affect their academic motivation (Emmanuel et al., 2014; Seaton et al., 2014), academic growth (Zhang et al., 2018), academic achievement (McInerney et al., 2012; Chao et al., 2019), and success (Prince and Nurius, 2014; Lohbeck et al., 2017). Accordingly, determining factors that may positively affect students’ academic self-concept seems essential. To respond to this necessity, several researchers have examined the impact of various personal (e.g., personality, sense of belonging) and interpersonal factors (e.g., teacher–student rapport, teacher support) on students’ academic self-concept (Curtin et al., 2013; Kim and Sax, 2014; McFarland et al., 2016; Trautwein and Möller, 2016; Cooper et al., 2018; Ma et al., 2021, to cite a few). Nevertheless, the influence of emotional and psychological factors such as positive mood and future outlook has remained elusive.

Generally, mood is a transient emotional state that is limited to a particular time and situation (Jeon, 1990). Positive mood also refers to “one’s mental state and feelings where she/he feels more confident, optimistic, and unconstrained” (Febrilia and Warokka, 2014, p. 3). As Brand et al. (2007) noted, students with positive moods are more likely to achieve academic success. Similarly, Cianci and Bierstaker (2009) also submitted that students’ positive mood enables them to achieve higher learning outcomes. Following such statements, some studies have been conducted on positive mood and its educational consequences (e.g., Lount, 2010; Akbari Chermahini and Hommel, 2012; Febrilia and Warokka, 2014; Grol and Raedt, 2014). As evidenced by the results of these studies, students’ positive mood is closely related to their increased achievement, higher learning outcomes, and success.

Future outlook as another influential factor in students’ academic self-concept pertains to one’s beliefs and expectations regarding the formation of future events (Nuttin, 2014). As Seginer (2009) maintained, students’ ideas of how well they will perform on forthcoming learning activities can favorably influence their academic performance. Schoon (2012) also asserted that students who firmly believe in a bright future are more likely to experience success in different aspects of their lives. Considering these assertions, several investigations have been conducted to determine the factors contributing to students’ positive future outlooks (Shane and Heckhausen, 2016; Schoon and Mortimer, 2017; Pax, 2020).

With regard to the principles of positive psychology, positive mental state and feelings that language learners experience in classroom contexts (i.e., positive mood) and the positive beliefs they possess about their future (i.e., future outlook) may favorably influence their academic behaviors (Dewaele et al., 2019; Li et al., 2020; Wang et al., 2021). Yet, not much attention has been paid to the impact of positive mood and future outlook on English as a foreign language (EFL/ESL) students’ academic behaviors. Moreover, to the best of the author’s knowledge, no empirical and review study has been conducted to delve into the impact of positive mood and future outlook on EFL students’ academic self-concept. To address these lacunas, the current review study seeks to examine the effects of positive mood and future outlook on EFL students’ academic self-concept.

## Review of Literature

### Positive Mood

Mood in a general sense pertains to “*one’s feeling state*” or how an individual feels when executing a particular activity (Fiedler et al., 2003). While there is a debate on how to categorize different types of moods (Djamasbi et al., 2010), in the realm of research, moods are typically grouped under three broad categories: negative, neutral, and positive (Venkatesh and Speier, 1999). The third category (i.e., positive mood), which is the focus of the present study, has been characterized as “one’s mental state and feelings where she/he feels more confident, optimistic, and unconstrained” (Febrilia and Warokka, 2014, p. 3). The concept of positive mood theoretically dates back to “positive mood theory” (Isen, 2008). Relying on this theory, being in a good mood can have a considerable impact on how individuals perceive themselves and others. It can also affect how one thinks about his/her own capabilities, abilities, and skills (Fredrickson, 2003; Djamasbi et al., 2009).

So far, some researchers have studied positive mood and its association with a range of educational factors (e.g., Akbari Chermahini and Hommel, 2012; Febrilia and Warokka, 2014; Grol and Raedt, 2014; Holman and Niven, 2019; Mackie and Worth, 2020; Wang and Guan, 2020). Febrilia and Warokka (2014), for instance, have probed into the impact of positive mood on students’ achievement and academic performance. To do so, three relevant questionnaires were given to 106 university students. The participants’ answers to the close-ended questionnaires were analyzed using structural equation modeling. The results of the analysis evinced that a positive mood has a favorable influence on both students’ achievement and academic performance. As another instance, Holman and Niven (2019) have examined the role of positive mood in students’ task performance. To gather the required data, two valid measures of the variables were administered to 78 college students. The inspection of students’ answers revealed that positive mood as an emotional trait can serve a facilitative function in improving students’ task performance.

### Future Outlook

Future outlook pertains to one’s beliefs and expectations regarding the formation of future events (Nuttin, 2014). Future outlook as a psychological construct encompasses two distinct components, namely future expectancy and future worry (Wigfield and Eccles, 2001). Future expectancy as the first component deals with “how one expects they will perform on upcoming tasks” (Eccles, 1983, p. 76). Future worry, on the other hand, relates to “the cognitive activity of worrying about the future” (Eccles and Wigfield, 1995, p. 216). In light of the expectancy-value theory, Wigfield et al. (2017) submitted that students’ beliefs and expectations about their academic future can affect their learning outcomes. To them, more positive beliefs and expectations will culminate in more desirable learning outcomes. Accordingly, one can conclude that students’ learning outcomes are subjected to their future outlook. Due to the importance of future outlook, some investigations have been carried out into this construct to uncover its educational consequences. A review of the existing literature indicates that students’ future outlook can drastically influence their academic achievement (Shane and Heckhausen, 2016; Schoon and Mortimer, 2017).

### Student Academic Self-Concept

Self-concept generally refers to a set of beliefs, ideas, and dispositions people maintain and describe themselves (Drew and Watkins, 1997). Further referred to this concept as a multi-faceted construct that represents individuals’ appraisal of their own abilities and skills. Extending this definition to the educational domain, Mercer (2011) defined student academic self-concept as individual students’ viewpoints regarding their academic capabilities and learning skills. As Mercer (2009) mentioned, student self-concept is a “*dynamic situational construct*” that may change over the course of life. Consistent with ideas of Mercer (2009), Weiner (2010) also submitted that students’ beliefs about themselves and their capabilities are alterable. To illustrate the value of student academic self-concept, Dörnyei (2009) maintained that how students see themselves can greatly influence their academic behaviors. Further, Huang (2011) also asserted that students’ self-concept can predict their success in educational contexts. More recently, Möller et al. (2020) stated that students’ academic self-concept is tied with their increased achievement. That is, any improvement in students’ academic self-concept may culminate in higher learning outcomes.

To date, several inquiries have been conducted on student academic self-concept, its antecedents (e.g., Franklin et al., 2017; Wolff et al., 2018; Yeboah et al., 2018; Kulakow, 2020; Biyikl, 2021, to cite a few), and its possible consequences (e.g., Chen et al., 2015; Amiryousefi and Mirkhani, 2019; Colmar et al., 2019; Ehm et al., 2019; Asadzadeh Maleki et al., 2021; Haktanir et al., 2021, to cite a few). Concerning the antecedents of student academic self-concept, Franklin et al. (2017), for instance, explored the factors predicting college students’ academic self-concept. To do so, 132 African American students were invited to take part in some interview sessions. The analysis of students’ answers to the interview questions demonstrated that student–professor interactions and grade point average are two significant predictors of students’ academic self-concept. By the same token, Yeboah et al. (2018) probed into the factors that may significantly promote high school students’ self-concept. To this end, 40 American students were interviewed in order to identify the determinants of students’ self-concept. The findings revealed that collaborative learning activities, teacher–student rapport, student–student interactions, and parental support can positively predict students’ academic self-concept.

To determine the consequences of student self-concept, Chen et al. (2015) have studied students’ academic self-concept in relation to their academic achievement. To this aim, two questionnaires were distributed among 407 college students. Inspecting the correlation of questionnaires, the researchers found that college students’ academic self-concept is closely related to their academic achievement. Similarly, Colmar et al. (2019) have delved into the role of students’ self-concept in improving their academic performance. In doing so, 191 students were asked to express their viewpoints by completing two valid questionnaires. They perceived student self-concept to be a favorable predictor of academic performance. In another study, Amiryousefi and Mirkhani (2019) have inspected the role of students’ academic self-concept in their willingness to communicate. To this aim, the questionnaires of willingness to communicate and academic self-concept were given to 136 Iranian students. The analysis of students’ viewpoints indicated that students’ willingness to communicate can be remarkably predicted by their academic self-concept.

### The Impact of Positive Mood and Future Outlook on EFL Students’ Academic Self-Concept

Drawing on positive psychology principles, Dewaele et al. (2019) asserted that the positive mental states that EFL students experience in English language classes may favorably affect their academic behaviors, including academic self-concept. Consistent with Dewaele et al. (2019) assertion, Li et al. (2020) also maintained that students’ positive moods caused by teachers, classmates, or the learning environment can positively influence their academic behaviors. Regarding the impact of future outlook on EFL students’ self-concept, Wang et al. (2021) stated that being optimistic about the future may have a positive impact on EFL students’ academic self-concept. That is, those EFL students who have positive beliefs and expectations about their academic future are able to improve their academic self-concept.

## Conclusion and Pedagogical Implications

So far, different definitions and conceptualizations of positive mood, future outlook, and student academic self-concept were offered. Relying on positive psychology principles, the favorable effects of positive mood and future outlook on EFL students’ academic self-concept were also illustrated. With regard to the theoretical evidence, one can fairly conclude that EFL students’ academic self-concept can be remarkably influenced by both positive mood and future outlook. This finding appears to be informative and instructive for EFL teachers in any educational environment. Given the importance of positive moods and emotions in improving students’ academic self-concept (Dewaele et al., 2019; Wang et al., 2021), teachers who are in charge of enhancing students’ academic behaviors should provide students with an enjoyable learning atmosphere in order to instill positive feelings in their minds. Moreover, as positive teacher interpersonal behaviors will result in students’ positive state of mind (Xie and Derakhshan, 2021), teachers are expected to make use of effective interpersonal behaviors in interactions with their pupils. The finding of this review seems to be illuminative for EFL students as well. As the findings of this review indicated, students’ beliefs and attitudes about their future can largely influence their academic self-concept. Students are thus required to be optimistic about their future in order to improve their academic self-concept. Given the paucity of research on positive mood, future outlook, and their interrelationships with EFL students’ academic self-concept, future investigations on this topic are thus recommended.

## Author Contributions

The author confirms being the sole contributor of this work and has approved it for publication.

## Conflict of Interest

The author declares that the research was conducted in the absence of any commercial or financial relationships that could be construed as a potential conflict of interest.

## Publisher’s Note

All claims expressed in this article are solely those of the authors and do not necessarily represent those of their affiliated organizations, or those of the publisher, the editors and the reviewers. Any product that may be evaluated in this article, or claim that may be made by its manufacturer, is not guaranteed or endorsed by the publisher.
